# Integrated clinical and laboratory assessment for severity classification in pediatric pertussis

**DOI:** 10.3389/fmed.2026.1961021

**Published:** 2026-10-21

**Authors:** Xuexin Wang, Xiaoping Zhu

**Affiliations:** 1 Pediatrics Department, Guizhou Medical University, Guizhou, China; 2 Pediatric Respiratory Department, The Affiliated Hospital of Guizhou Medical University, Guizhou, China

**Keywords:** admission-time classification, children, disease severity, hypoalbuminemia, leukocytosis, pertussis, pneumonia, severity classification

## Abstract

**Background:**

Early identification of children with severe pertussis is essential for timely intervention and improved clinical outcomes.

**Objective:**

To assess clinical and laboratory characteristics associated with severe pertussis and to evaluate their ability to classify severe disease at admission among hospitalised children.

**Methodology:**

This retrospective cohort study included 722 children aged 1 month–13 years with laboratory-confirmed pertussis admitted from January 2020 to December 2025. Clinically diagnosed, laboratory-unconfirmed cases were excluded. Demographic, clinical, laboratory, radiological, and outcome data were extracted from electronic medical records. Univariable and multivariable logistic regression identified factors associated with severe pertussis, with model performance assessed by AUC. Because several predictors contributed to the definition of severe disease, the integrated model was interpreted as a concurrent classification model. A second model using demographic, historical, and admission laboratory variables was interpreted as an admission-time severity classification model. Bootstrap internal validation and calibration were performed.

**Results:**

Severe pertussis occurred in 157 children (21.7%). In the concurrent model, apnea and respiratory distress were strongly associated with severe disease but were not interpreted as independent predictors because they formed part of the severity definition. Age <6 months, prematurity, incomplete-for-age or absent vaccination, radiological pneumonia, marked leukocytosis, hypoalbuminemia, and low immunoglobulin G (IgG) levels were also independently associated with severe disease (all *p* < 0.05). The apparent AUC was 0.89 (sensitivity 83.4%, specificity 82.1%, accuracy 82.4%), although this performance may be overestimated because of outcome-information leakage. In the admission-time model, age <6 months, incomplete-for-age or absent vaccination, higher white blood cell and absolute lymphocyte counts, and lower albumin and IgG levels were independently associated with severe pertussis. For the admission-time model, AUC was 0.961 (95% CI 0.947–0.975), with an optimism-corrected figure of 0.957, an apparent measure of discrimination that should not be taken as a measure of prediction of clinical deterioration.

**Conclusion:**

Severe pertussis was associated with distinct demographic and laboratory abnormalities, with admission-time variables showing strong discrimination for severe disease. Given that many severe cases met severity criteria at the index event, the admission-time model should not be interpreted as prognostic for subsequent deterioration.

## 1 Introduction

Pertussis, also known as whooping cough, is a highly contagious, vaccine-preventable respiratory illness caused by the bacterium *Bordetella pertussis.* While it is recognised as a disease which is preventable, it is an important cause of morbidity and mortality in infants and young children in the world, especially where immunisation is not achieved. Many outbreaks still occur; incomplete vaccine coverage, delayed diagnosis, and declining vaccine-induced immunity remain a continued burden, especially in low- and middle-income countries. Infants under 6 months are most susceptible because they may not be fully immunised and their immune systems are not fully developed (1). Pertussis can often result in hospitalisation, particularly if it is a severe case and requires intensive care. Apnea, pneumonia, respiratory failure, pulmonary hypertension, seizures, encephalopathy and death are among the common complications (2, 3). In previous studies, several demographic and clinical features, such as low birth weight, prematurity, malnutrition, delayed health service presentation, and incomplete vaccination, were associated with severe disease (4).

Clinical manifestations of pertussis in children can range from mild catarrhal symptoms to life-threatening respiratory illness, suggesting that severity depends on several clinical and laboratory factors. Several clinical factors, such as younger age, incomplete vaccination, prematurity, recurrent apnea, persistent paroxysmal cough, cyanosis, feeding difficulties, pneumonia, pulmonary hypertension, and neurological disorders, have been associated with severe disease or poor clinical outcomes in previous studies (5). Laboratory findings such as leukocytosis, lymphocytosis, elevated inflammatory markers and thrombocytosis, as well as abnormal arterial blood gas findings, have been associated with severe disease. In an autopsy series of fatal infant pertussis, hyperleukocytosis has been associated with pulmonary vascular obstruction, pulmonary hypertension and refractory respiratory failure (6). Hyperleukocytosis is thought to increase blood viscosity and impede the pulmonary microvasculature, resulting in a worse prognosis. Thus, laboratory biomarkers are useful alongside clinical assessment to enable earlier disease detection and intervention (7).

A comprehensive assessment is also needed for severe pertussis, based on its pathophysiology. B. pertussis virulence factors, including pertussis toxin, adenylate cyclase toxin, and tracheal cytotoxin, suppress the host immune response and damage the respiratory epithelium, driving disease progression. Pertussis toxin causes significant lymphocytosis and hyperleukocytosis, which can lead to pulmonary hypertension and cardiopulmonary complications (8), while adenylate cyclase toxin inhibits neutrophil and macrophage function and decreases bacterial clearance. These mechanisms are largely derived from experimental and pathological studies (7, 8); in routine clinical practice, laboratory abnormalities may reflect disease progression and, when used with clinical signs, may help identify severe cases early (9).

Several studies have examined clinical parameters or laboratory markers associated with severe pediatric pertussis, but few studies have evaluated their combined discriminatory performance. To analyse the association of demographic, vaccination, clinical, radiological, haematological, biochemical, and immunological parameters with severe pertussis and to identify factors linked to established severe disease versus factors that could be obtained at admission, the present study combined all these parameters. Special emphasis was placed on the time constraints of retrospective classification and interpretation of clinically important outcomes as independent prognostic factors. The aim of the present study was therefore to determine the clinical and laboratory characteristics associated with severe pertussis and to evaluate an admission-time severity classification model based on demographic, historical, and admission laboratory variables.

## 2 Methodology

### 2.1 Study design and setting

This retrospective cohort study was conducted at a tertiary paediatric referral hospital to examine integrated clinical and laboratory characteristics associated with pertussis severity and to evaluate their discriminatory performance for severity classification. The study was based on the most recent paediatric studies on demographic characteristics, clinical signs, haematological values, inflammatory markers, and factors predicting pertussis severity. This study was reported in accordance with the Strengthening the Reporting of Observational Studies in Epidemiology (STROBE) guidelines (10), and the classification analyses were reported with reference to the applicable TRIPOD statement (11). All participants had laboratory-confirmed pertussis; no non-pertussis comparison group was included, and all comparisons were made between severity groups within the pertussis cohort.

### 2.2 Study period and participant selection

This retrospective cohort study included infants and children admitted with pertussis from 1 January 2020 to 31 December 2025. Eligible participants were identified using electronic medical records from the hospital, admission database, ward discharge database, and microbiology laboratory register. A total of 864 medical records of children with suspected or documented pertussis were identified and reviewed for eligibility; only children with laboratory-confirmed pertussis were retained.

A total of 142 records were excluded during screening. Of these, 52 were excluded because pertussis was not laboratory-confirmed or another diagnosis was established; 24 because the child was outside the eligible age range; 18 because they were duplicate or repeat admissions for the same episode (so that each child contributed only one index admission); 30 because essential clinical, laboratory or severity data were missing; and 18 because documentation was insufficient to confirm eligibility. Of the 722 children with laboratory-confirmed pertussis, 565 (78.3%) had non-severe pertussis, and 157 (21.7%) met at least one predefined criterion for severe pertussis. Figure 1 describes the participant selection process. Basic clinical and laboratory characteristics of the excluded records were not retained in the final analysis dataset; therefore, a formal comparison between excluded and included patients could not be performed.

**Figure 1:**
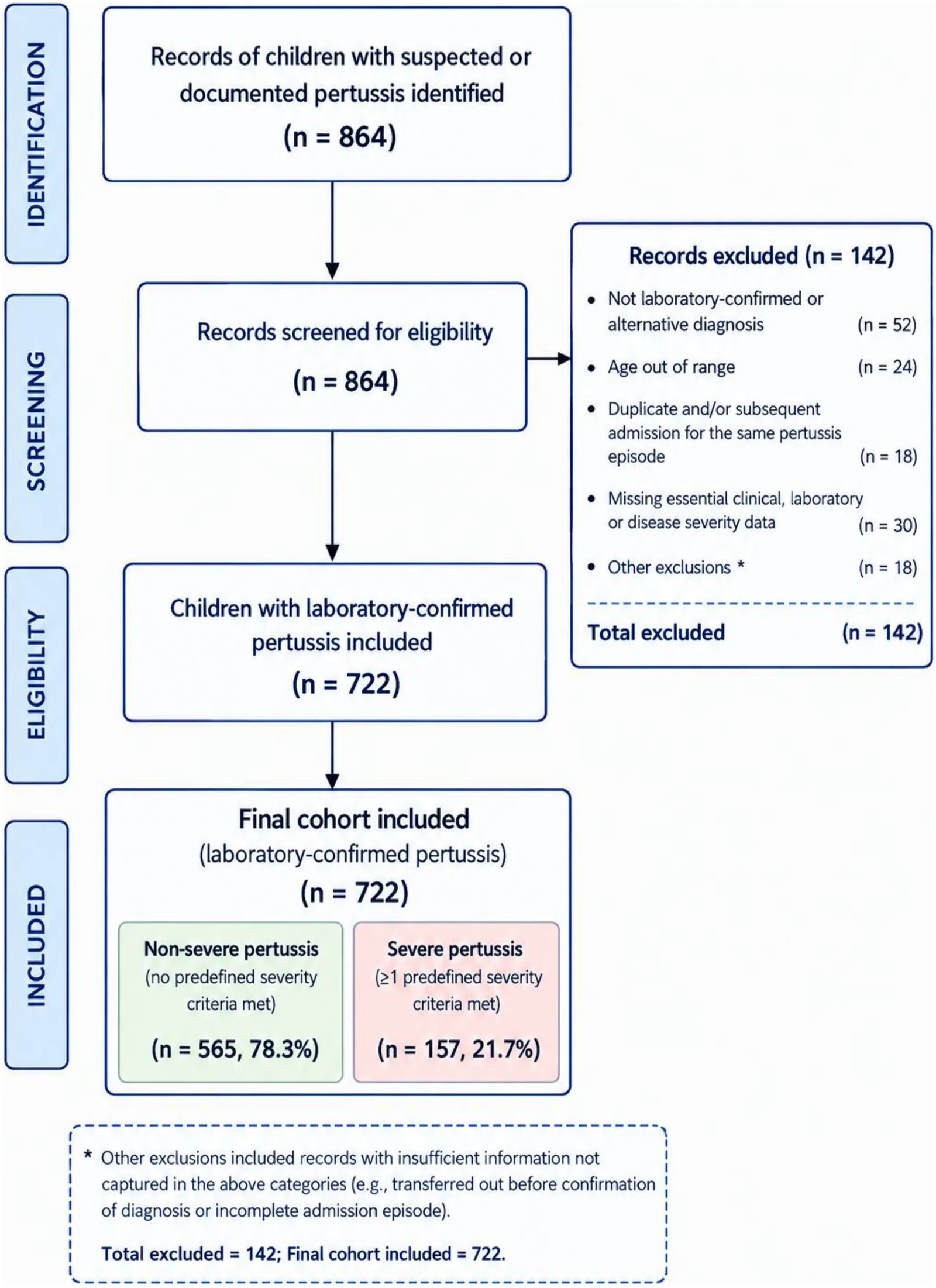
Participant selection flow diagram.

The flow diagram illustrates the identification, screening, exclusion, and final inclusion of children with laboratory-confirmed pertussis. The final cohort consisted of 722 patients, categorised into non-severe and severe pertussis groups according to the predefined composite severity criteria.

### 2.3 Eligibility criteria

Children were eligible to participate if they were between 1 month and 13 years of age, had laboratory-confirmed pertussis, were hospitalised during the study period, and had sufficient clinical and laboratory data to assess disease severity. Laboratory confirmation was defined as documented microbiological evidence of *Bordetella pertussis* in the hospital microbiology record. PCR detection of *B. pertussis* from an appropriate respiratory specimen was the principal laboratory confirmation method used during the study period; however, the available analysis dataset did not retain the individual confirmation method for each included patient (12). Children with a clinical diagnosis but without laboratory confirmation were excluded.

Patients who did not meet the criteria for confirmation of pertussis, were outside the age range, or were duplicate admissions for the same disease episode were excluded. Patients for whom data about the primary outcome or the relevant baseline clinical and laboratory data were missing were also excluded.

### 2.4 Clinical and demographic data collection

A standardised data collection form was used to extract data from electronic medical records, inpatient charts, laboratory databases, radiological reports, and discharge summaries. Demographic factors included age, sex, body weight, prematurity, underlying medical conditions and pertussis vaccination status. Age was recorded as chronological age at admission; corrected age for prematurity was not used. Vaccination status was classified as age-appropriate, incomplete-for-age, or unvaccinated according to the number of pertussis-containing vaccine doses expected for the child's chronological age under the national immunization schedule applicable during the study period. Immunisation information was collected from the documented immunisation in the medical record.

Clinical variables included cough duration before admission (the number of days from cough onset to hospital admission, as recorded in the admission history), paroxysmal cough, whoop on inspiration, vomiting after coughing, fever, apnea, cyanosis, feeding difficulty, respiratory distress, tachypnea, tachycardia, and abnormal respiratory examination findings. Data on pneumonia, intensive care unit admission, oxygen therapy, noninvasive respiratory support, invasive mechanical ventilation, length of hospital stay and clinical outcomes were also collected.

### 2.5 Laboratory and radiological assessment

Laboratory values were taken from the first blood sample obtained at admission, before escalation of respiratory support or other major therapeutic measures. For the admission-based analysis, the collection time of this first admission blood sample was set as the index time. The index time defined the temporal boundary for the admission-time classification analysis; it was not used to establish a prospective prognostic window for subsequent severe events. Laboratory parameters included total white blood cell count, absolute lymphocyte count, absolute neutrophil count, haemoglobin, platelet count, C-reactive protein, procalcitonin, serum albumin and routine biochemical parameters. Absolute neutrophil and lymphocyte counts were used to calculate the neutrophil-to-lymphocyte ratio.

Absolute lymphocyte count (ALC) is in ×10⁹/L and was derived from the complete blood count done on the same admission blood sample used to measure total WBC count. In checking data quality, WBC and ALC values were compared to the original differential blood-count record and patient identity and the time of the blood samples were also checked, along with the units used to measure the differential blood counts and the correspondence between the total WBC and differential counts. Thirty-four records had an ALC that was higher than the total WBC, which was biologically implausible and was thought to be data entry or variable-alignment errors on data abstraction. The values have been rechecked with the original laboratory report for the same sample and date of admission, and the values were corrected. These adjustments did not exclude any participants and the analytic sample consisted of 722 children. The other laboratory variables were similarly subject to a quality-control process at the patient level, such as checking the units, timeliness of the collection, checking the source record for consistency and checking for biologically unrealistic pre-analysis values.

Age-appropriate institutional reference ranges were used to interpret leukocytosis and lymphocytosis. Nutritional and protein status was assessed using serum albumin, which was categorised based on the pediatric laboratory reference value (LRV). Hypoalbuminemia was determined with the age-specific lower reference limits for serum albumin for the institution: [<36 g/L (1–<12 months) and <38 g/L (1–<13 years)]. The level of albumin was measured in the same blood sample taken on first hospital admission before any in-hospital fluid therapy or albumin/blood product was administered. Serum IgG was measured in all 722 children. Because IgG in young infants is largely maternally derived and declines physiologically during the first months of life (13), low IgG was defined using age-specific reference intervals. Radiological data from chest x-rays, such as atelectasis, peribronchial changes, consolidation and pulmonary infiltrates, were extracted from the radiology reports.

### 2.6 Outcome assessment and operational definitions

The main outcome was severe pertussis. The definition of severe disease was that the child had at least one major adverse clinical event in the hospital, such as admission to the intensive care unit, significant respiratory distress, clinically significant apnea or cyanosis, respiratory failure, persistent hypoxemia, need for noninvasive or invasive mechanical ventilation, hemodynamic instability, severe pulmonary or neurologic complications, or death while hospitalised. Children without these features were classified as having non-severe pertussis. Apnea, respiratory distress, cyanosis and hypoxemia are also part of this definition and any relationship of these with severity depends on the classification rule; these are considered as markers of established severe disease rather than as risk factors or predictors.

The clinical signs that were reported as part of the composite outcome (Table 2) and the components of the composite outcome were defined separately to ensure the classification was reproducible. Supplementary Table S2 presents the frequency and overlap of the individually recorded components of the composite severe-pertussis outcome among the 157 severe cases. Components that were not separately retained as binary variables in the final analysis dataset were not reconstructed from aggregate information. Outcome components were only counted if the more stringent severity criteria were met: clinically significant apnea was defined as either recurrent apnea (≥2 episodes in 24 h) or any apnea requiring stimulation, bag-mask ventilation or escalation of respiratory support; clinically significant respiratory distress as being associated with SpO₂ < 92% or requiring intervention; clinically significant cyanosis as cyanosis with SpO₂ < 92% [or requiring intervention]; and persistent hypoxemia as SpO₂ < 92% [sustained for ≥5 min or on ≥2 consecutive measurements despite supplemental oxygen]. Children with documented signs in Table 2 but who did not reach the corresponding outcome threshold (non-severe) had apnea (*n* = 67), respiratory distress (*n* = 96), cyanosis (*n* = 36), and SpO₂ < 92% (*n* = 24). A patient-level audit was done on all non-severe children with any of these signs to verify that no patient met the outcome threshold (no patient was reclassified from non-severe to severe) and no non-severe patient met the more stringent outcome threshold.

For the admission-based analysis, the index time was the collection of the first blood sample. The medical record was then checked for every child diagnosed with severe pertussis to see if any of the severe pertussis criteria had been met at or before the index time, or if a first diagnosis of severe pertussis occurred after the index time. Because the retrospective records did not permit reliable reconstruction of the exact timing of every severe criterion relative to the index time, the admission-time analysis was interpreted as a severity classification analysis rather than a prognostic analysis. Importantly, it should be noted that the index-time classification does not mean that all those who were considered severe had severe outcomes at or after the first blood sample taken, but rather that the index-time classification measures any difference in discrimination between those who had severe outcomes at or after the first blood sample and those who did not.

Prematurity was defined as being born before 37 weeks of gestation (14). Incomplete-for-age vaccination was defined as having received fewer pertussis-containing vaccine doses than expected for the child's age according to the national immunisation schedule applicable during the study period. Unvaccinated children had no documented receipt of pertussis-containing vaccine doses. Apnea was defined as a documented stoppage in breathing with oxygen desaturation, cyanosis, bradycardia, or clinical intervention. Respiratory distress was defined as an increase in work of breathing (chest retractions, nasal flaring, grunting, or age-specific tachypnea). Pneumonia was classified as meeting the following criteria: compatible respiratory symptoms and/or radiologic pulmonary infiltration or consolidation (15). Criteria for leukocytosis and lymphocytosis were based on the laboratory reference range for each age group, and hypoalbuminemia was defined as serum albumin less than the institutional pediatric reference range (16).

### 2.7 Statistical analysis

IBM Statistical Package for the Social Sciences (SPSS) version 27.0 (IBM Corp., Armonk, NY, United States) was used to perform statistical analyses; variance inflation factors, the Hosmer–Lemeshow test, DeLong confidence intervals and bootstrap internal validation for the admission-based model were computed in Python (statsmodels and scikit-learn). The Shapiro–Wilk test was used to assess normality in continuous variables. Normally distributed variables were presented as mean ± SD and compared using an independent-samples t-test. Non-normally distributed data were presented as median and interquartile range and compared using the Mann–Whitney U test. Categorical variables were described as frequencies and percentages, and statistical comparisons were performed using the chi-square test or Fisher's exact test.

Univariable logistic regression was used to examine associations between demographic, clinical, laboratory, and radiological characteristics and severe pertussis. Multivariable logistic regression was used to estimate adjusted associations and to classify established severe disease. The model is not intended to identify independent risk factors or predictors; rather, it classifies established severe disease as part of the outcome definition, and the odds ratios and AUC are partly dependent on the classification rule and are therefore likely inflated by outcome-information leakage.

To separate characteristics present at admission from features of established severe disease, we created an additional multivariable logistic regression model that included these baseline characteristics at admission. This analysis was limited to variables measured at initial evaluation and did not include measures of clinical deterioration or its effects. Age below 6 months, sex, prematurity, vaccination status, underlying comorbidity, white blood cell (WBC) count, absolute lymphocyte count, serum albumin, and serum IgG were considered as candidate variables. These predictors were prespecified on the basis of clinical relevance, prior literature, and their temporal availability at the index time rather than being selected solely according to univariable p-values. Univariable analyses were used to describe individual associations and were not used as a statistical screening criterion for entry into the admission-time multivariable model.

Other in-hospital variables, such as apnea, respiratory distress, respiratory failure, intensive care unit (ICU) admission, respiratory support and other in-hospital parameters, were not included in this admission-based model as they may represent already developed or progressive severe disease. Continuous laboratory variables were kept continuous in order not to lose information due to arbitrary categorisation. In the primary integrated model, laboratory variables were entered in their clinically used dichotomised form to reflect bedside interpretation, which partly explains the difference in discrimination between the two models. Odds ratios (ORs) with 95% confidence intervals (CIs) and p-values were computed. Area under the receiver operating characteristic curve (AUC) was used to measure the performance of the models with respect to discrimination. This model used laboratory measurements from the first admission blood sample (index time). This is because some children may have already met one or more severe-pertussis criteria at that time and are therefore not considered to be a model predicting subsequent deterioration, but rather an admission-time severity classification model.

Results were presented as crude and adjusted odds ratios (ORs) and 95% CI. Multicollinearity was measured by variance inflation factors (VIFs), with values >5 considered problematic, especially for absolute lymphocyte count (ALC) because lymphocytes are part of the WBC. The Hosmer–Lemeshow goodness-of-fit test was used to assess model calibration, and the area under the receiver operating characteristic curve was used to assess model discrimination. Because the admission-based model was developed and evaluated in the same cohort, it was internally validated with 1,000 bootstrap resamples, and an optimism-corrected AUC was reported (17); the 95% CI of its AUC was calculated by the DeLong method (18). In a sensitivity analysis, the admission-based model was refitted with additional adjustment for cough duration before admission (days from cough onset to admission; admission blood samples were taken on the day of admission), because illness duration at sampling may influence laboratory values. Additional analyses of the model based on the admission were conducted. First, the correlation of WBC and ALC was tested using both Pearson and Spearman correlation coefficients, overall and by age groups (younger children <6 months, 6–11 months, 1–4 years, 5–13 years). Second, the coefficients for WBC and ALC are conditional, since they are entered in the same block, and because ALC is a subcategory of WBC. Thus, the sensitivity models were fitted using only WBC, only ALC, and ALC and non-lymphocyte WBC (WBC minus ALC) to evaluate the robustness and interpretation of these associations. Third, to test the linearity of the continuous predictors on the logit scale, restricted cubic splines were used, and age was also modelled as a continuous variable using restricted cubic splines instead of just the 6-month cut-off. The IgG association may have been influenced by age; therefore, a sensitivity analysis was performed with age-specific low-IgG status (defined as the institutional low-IgG concentration by age) and compared to the primary model, which was continuous IgG.

To assess the performance of a simpler model without IgG, we fitted a separate sensitivity model using the same admission-time covariates as the primary model but excluding IgG. Discrimination was measured by the AUC with DeLong 95% CI and calibration was measured by the calibration intercept and slope. An optimism-corrected AUC was reported, and 1,000 bootstrap resamples were performed to measure internal optimism. Fourth, calibration was evaluated using a calibration plot and calibration intercept and slope (apparent optimisation and bootstrap), since the Hosmer–Lemeshow test is not sufficient. Fifth, to examine the confounding by the albumin association, the model was re-estimated with further adjustment for C-reactive protein, feeding difficulty, alanine aminotransferase and serum creatinine. Because models that include outcome-defining variables do not assess the same prognostic performance as the admission-based model, AUCs are not compared with paired tests, and no superiority of one model over the other is claimed.

### 2.8 Ethical considerations

The Ethics Committee of Guizhou Medical University approved this research (Approval Number: 2026-032). The study was carried out in accordance with the ethical rules of the Declaration of Helsinki and its amendments (19). Only authorised researchers accessed the study data, and study information was used only for research purposes.

## 3 Results

### 3.1 Demographic characteristics, perinatal history, and baseline clinical profile

Table 1 summarises the demographic and baseline characteristics of the study population. Children with severe pertussis were younger than those with non-severe disease (4.0 [2.0–10.0] vs 12.0 [5.0–34.0] months; *p* < 0.001), and children aged <6 months comprised 64.3% of those with severe disease vs 29.6% with non-severe disease (*p* < 0.001). Children with severe disease were also more likely to be premature and to have low birth weight (21.0% vs 8.3%; *p* < 0.001 and 14.6% vs 7.1%; *p* = 0.003, respectively). Less age-appropriate vaccination was seen amongst severe cases (32.5% vs 60.7%, *p* < 0.001), while incomplete or no vaccination was more common. The severe group also had a higher prevalence of underlying comorbidities (24.2% vs. 12.2%, *p* < 0.001), with congenital heart disease, chronic respiratory disease and neurological disorders more common. No statistically significant difference in the proportion of males was detected between the groups (*p* = 0.401).

**Table 1:** Demographic, perinatal, and baseline characteristics according to pertussis severity.

Variable	Overall (*n* = 722)	Non-severe (*n* = 565)	Severe (*n* = 157)	*p*-value
Age, months, median (IQR)	9.0 (4.0–28.0)	12.0 (5.0–34.0)	4.0 (2.0–10.0)	<0.001
Age <6 months	268 (37.1)	167 (29.6)	101 (64.3)	<0.001
Age 6–11 months	151 (20.9)	124 (21.9)	27 (17.2)	0.193
Age 1–4 years	205 (28.4)	184 (32.6)	21 (13.4)	<0.001
Age 5–13 years	98 (13.6)	90 (15.9)	8 (5.1)	0.001
Male sex	392 (54.3)	302 (53.5)	90 (57.3)	0.401
Female sex	330 (45.7)	263 (46.5)	67 (42.7)	0.401
Premature birth	80 (11.1)	47 (8.3)	33 (21.0)	<0.001
Low birth weight history	63 (8.7)	40 (7.1)	23 (14.6)	0.003
Age-appropriate vaccination	394 (54.6)	343 (60.7)	51 (32.5)	<0.001
Incomplete-for-age vaccination	294 (40.7)	202 (35.8)	92 (58.6)	<0.001
Unvaccinated	34 (4.7)	20 (3.5)	14 (8.9)	0.004
Any underlying comorbidity	107 (14.8)	69 (12.2)	38 (24.2)	<0.001
Congenital heart disease	31 (4.3)	17 (3.0)	14 (8.9)	0.002
Chronic respiratory disease	42 (5.8)	27 (4.8)	15 (9.6)	0.024
Neurological disorder	19 (2.6)	11 (1.9)	8 (5.1)	0.027

Data are presented as *n* (%) unless otherwise stated. IQR, interquartile range. *p*-values were calculated using the Mann–Whitney *U* test for age and the chi-square or Fisher's exact test for categorical variables. A two-sided *p*-value <0.05 was considered statistically significant. Comparisons are descriptive and were not adjusted for multiple testing.

### 3.2 Clinical manifestations and respiratory findings

Table 2 summarises clinical manifestations and respiratory examination findings by disease severity. Several of these features (apnea, cyanosis, respiratory distress, and hypoxemia) are also components of the severity definition, so their higher frequency in the severe group partly reflects the classification rule. Some non-severe children have these as part of the children's general descriptive definitions, rather than the outcome thresholds (Section 2.6). Severe pertussis was characterised by a substantially higher frequency of apnea (51.6% vs. 11.9%, *p* < 0.001), cyanosis (38.2% vs. 6.4%, *p* < 0.001), feeding difficulty (55.4% vs. 22.1%, *p* < 0.001), and respiratory distress (70.1% vs. 17.0%, *p* < 0.001). Age-adjusted tachypnea and tachycardia were also significantly more common among severe cases (63.7% vs 24.6% and 46.5% vs 19.1%, respectively; both *p* < 0.001). Severe cases had oxygen saturations <92% in 51.0%, whereas non-severe cases had oxygen saturations <92% in 4.2% (*p* < 0.001). The severe group also had significantly higher rates than non-severe group for chest retractions, moist rales/crackles, reduced breath sounds, and altered mental status (all *p* < 0.001). No statistically significant differences were detected in paroxysmal cough, inspiratory whoop, post-tussive vomiting, or wheezing.

**Table 2:** Clinical manifestations and examination findings according to disease severity.

Clinical variable	Overall (*n* = 722)	Non-severe (*n* = 565)	Severe (*n* = 157)	*p*-value
Cough duration before admission, days, median (IQR)	12 (9–16)	12 (9–16)	13 (9–15)	0.566
Paroxysmal cough	612 (84.8)	482 (85.3)	130 (82.8)	0.447
Inspiratory whoop	269 (37.3)	221 (39.1)	48 (30.6)	0.052
Post-tussive vomiting	228 (31.6)	185 (32.7)	43 (27.4)	0.204
Fever	204 (28.3)	143 (25.3)	61 (38.9)	0.001
Apnea†	148 (20.5)	67 (11.9)	81 (51.6)	<0.001
Cyanosis†	96 (13.3)	36 (6.4)	60 (38.2)	<0.001
Feeding difficulty	212 (29.4)	125 (22.1)	87 (55.4)	<0.001
Respiratory distress†	206 (28.5)	96 (17.0)	110 (70.1)	<0.001
Age-adjusted tachypnea	239 (33.1)	139 (24.6)	100 (63.7)	<0.001
Age-adjusted tachycardia	181 (25.1)	108 (19.1)	73 (46.5)	<0.001
Chest retractions	176 (24.4)	79 (14.0)	97 (61.8)	<0.001
Oxygen saturation <92%†	104 (14.4)	24 (4.2)	80 (51.0)	<0.001
Wheezing	187 (25.9)	143 (25.3)	44 (28.0)	0.504
Moist rales/crackles	243 (33.7)	157 (27.8)	86 (54.8)	<0.001
Reduced breath sounds	75 (10.4)	31 (5.5)	44 (28.0)	<0.001
Altered mental status	29 (4.0)	6 (1.1)	23 (14.6)	<0.001

Data are presented as *n* (%). IQR, interquartile range. Tachypnea and tachycardia were classified using age-adjusted pediatric reference values. † Variable overlaps with a component of the severe-pertussis definition; comparisons are therefore partly circular and are shown descriptively. Table 2 variables are described with the purpose of being broader than the thresholds used for the outcome component that it corresponds to (see Section 2.6) and may not have a registered sign if the child is not a non-severe child.

### 3.3 Haematological, inflammatory, biochemical, and immunological findings

Table 3 summarises the haematological, inflammatory, biochemical, and immunological results. Children with severe pertussis had higher median white blood cell counts (24.8 vs 15.7 × 10⁹/L, *p* < 0.001), absolute lymphocyte counts (12.4 vs 8.2 × 10⁹/L, *p* < 0.001), and absolute neutrophil counts (8.1 vs 5.5 × 10⁹/L, *p* < 0.001) than those with non-severe disease. Leukocytosis and lymphocytosis were significantly more frequent with severe illness (43.3% vs 8.1%, *p* < 0.001 and 54.1% vs 21.1%, *p* < 0.001, respectively). Lower haemoglobin and serum albumin levels, and higher platelet counts, C-reactive protein, procalcitonin, and alanine aminotransferase levels were also associated with severe disease (all *p* < 0.001). Hypoalbuminemia was seen in 42.0% of severe cases versus 11.7% of non-severe cases (*p* < 0.001). Reduced IgG (below the age-specific reference interval) was also more common in severe disease (28.0% vs. 11.0%, *p* < 0.001).

**Table 3:** Laboratory and immunological characteristics according to pertussis severity.

Laboratory variable	Overall (*n* = 722)	Non-severe (*n* = 565)	Severe (*n* = 157)	*p*-value
White blood cell count, ×10⁹/L	17.4 (12.1–24.2)	15.7 (11.4–20.8)	24.8 (18.5–34.1)	<0.001
Marked leukocytosis	114 (15.8)	46 (8.1)	68 (43.3)	<0.001
Absolute lymphocyte count, ×10⁹/L	9.1 (6.1–13.2)	8.2 (5.7–11.4)	12.4 (8.3–17.0)	<0.001
Lymphocytosis	204 (28.3)	119 (21.1)	85 (54.1)	<0.001
Absolute neutrophil count, ×10⁹/L	6.0 (3.9–8.9)	5.5 (3.6–7.9)	8.1 (5.5–11.6)	<0.001
Neutrophil-to-lymphocyte ratio	0.66 (0.43–0.98)	0.62 (0.41–0.90)	0.76 (0.48–1.18)	<0.001
Haemoglobin, g/L	108 ± 15	110 ± 14	101 ± 17	<0.001
Anemia	161 (22.3)	105 (18.6)	56 (35.7)	<0.001
Platelet count, ×10⁹/L	389 (305–477)	376 (298–457)	432 (334–542)	<0.001
C-reactive protein, mg/L	8.4 (3.1–20.5)	6.7 (2.7–15.4)	17.8 (7.0–39.2)	<0.001
Elevated C-reactive protein	276 (38.2)	184 (32.6)	92 (58.6)	<0.001
Procalcitonin, ng/mL	0.16 (0.07–0.39)	0.12 (0.06–0.28)	0.39 (0.15–1.04)	<0.001
Serum albumin, g/L	38.2 ± 4.9	39.1 ± 4.4	34.8 ± 5.1	<0.001
Hypoalbuminemia	132 (18.3)	66 (11.7)	66 (42.0)	<0.001
Alanine aminotransferase, U/L	28 (19–42)	26 (18–38)	36 (23–59)	<0.001
Serum creatinine, µmol/L	31 (24–39)	31 (24–38)	33 (25–42)	0.018
Immunoglobulin G, g/L	6.8 (5.1–8.5)	7.1 (5.4–8.8)	5.8 (4.4–7.5)	<0.001
Reduced immunoglobulin G	106 (14.7)	62 (11.0)	44 (28.0)	<0.001

Data are presented as median (IQR), mean ± standard deviation, or *n* (%). Laboratory abnormalities were classified using age-adjusted institutional pediatric reference ranges. Comparisons are descriptive and not adjusted for multiple testing.

### 3.4 Radiological findings, clinical complications, and hospital outcomes

The results of the radiological evaluations, clinical complications, treatment needs and hospital outcomes are summarised in Table 4. The manifestations and outcomes of severe disease recorded at the individual level in the 157 children with pertussis classified as severe were: Respiratory distress, respiratory failure, admission to pediatric intensive care, apnea, oxygen saturation <92%, invasive mechanical ventilation, noninvasive respiratory support, pulmonary hypertension, neurological complications, and in-hospital mortality. A number of the individual components may overlap in one patient; distributions and frequencies of individual components relating to severe disease are shown in Supplementary Table S2.

**Table 4:** Radiological findings, complications, treatment requirements, and outcomes.

Variable	Overall (*n* = 722)	Non-severe (*n* = 565)	Severe (*n* = 157)	*p*-value
Any abnormal chest imaging	313 (43.4)	201 (35.6)	112 (71.3)	<0.001
Radiological pneumonia	220 (30.5)	130 (23.0)	90 (57.3)	<0.001
Pulmonary consolidation	116 (16.1)	49 (8.7)	67 (42.7)	<0.001
Atelectasis	67 (9.3)	24 (4.2)	43 (27.4)	<0.001
Multilobar involvement	49 (6.8)	13 (2.3)	36 (22.9)	<0.001
Respiratory failure	91 (12.6)	0 (0.0)	91 (58.0)	<0.001
Pediatric intensive care admission	126 (17.5)	0 (0.0)	126 (80.3)	<0.001
Supplemental oxygen	203 (28.1)	72 (12.7)	131 (83.4)	<0.001
Noninvasive respiratory support	48 (6.6)	0 (0.0)	48 (30.6)	<0.001
Invasive mechanical ventilation	55 (7.6)	0 (0.0)	55 (35.0)	<0.001
Pulmonary hypertension	22 (3.0)	1 (0.2)	21 (13.4)	<0.001
Neurological complications	24 (3.3)	4 (0.7)	20 (12.7)	<0.001
Length of hospital stay, days	8 (6–12)	7 (5–10)	15 (10–23)	<0.001
In-hospital mortality	13 (1.8)	0 (0.0)	13 (8.3)	<0.001

Data are presented as *n* (%) or median (IQR). Intensive care admission, respiratory failure, and advanced respiratory support were components of the predefined severe pertussis outcome; comparisons of these variables are descriptive and follow from the classification rule.

Abnormal chest imaging was significantly more common in children with severe pertussis than in those with non-severe disease (71.3% vs. 35.6%, *p* < 0.001). It included radiological pneumonia (57.3% vs. 23.0%, *p* < 0.001), pulmonary consolidation (42.7% vs. 8.7%, *p* < 0.001), atelectasis (27.4% vs. 4.2%, *p* < 0.001), and multilobar involvement (22.9% vs. 2.3%, *p* < 0.001). Severe cases more often required supplemental oxygen (83.4% vs. 12.7%, *p* < 0.001); noninvasive respiratory support (30.6% vs. 0%, *p* < 0.001); and invasive mechanical ventilation (35.0% vs. 0%, *p* < 0.001). Because respiratory failure, PICU admission and advanced respiratory support are components of the severity definition, these comparisons are descriptive. In-hospital mortality was also higher in severe cases (8.3% vs 0%), as was the median hospital stay (15 days vs 7 days, *p* < 0.001).

### 3.5 Univariable logistic regression analysis of factors associated with severe pertussis

Several demographic, clinical, laboratory and radiological factors were found to be significantly associated with severe pertussis in the univariable logistic regression analysis (Table 5). Age (<6 months) was significantly associated with severe disease, with odds more than four times higher (OR 4.27, 95% CI 2.91–6.27, *p* < 0.001); and underlying comorbidities were also associated with severe disease (OR 2.30, 95% CI 1.47–3.59, *p* < 0.001). The largest associations were observed for clinical indicators, particularly oxygen saturation <92% (OR 23.52, 95% CI 13.94–39.68), respiratory distress (OR 11.40, 95% CI 7.59–17.11), cyanosis (OR 9.04, 95% CI 5.55–14.72), and apnea (OR 7.91, 95% CI 5.25–11.93), all with *p* < 0.001; however, these variables overlap with the severity definition, and their large ORs largely reflect that overlap. Additionally, laboratory abnormalities such as marked leukocytosis, lymphocytosis, increased C-reactive protein, hypoalbuminemia, decreased immunoglobulin G, and anaemia were significantly associated with severe pertussis.

**Table 5:** Univariable logistic regression analysis of potential predictors of severe pertussis.

Variable	Crude OR	95% CI	*p*-value
Age <6 months	4.27	2.91–6.27	<0.001
Male sex	1.17	0.82–1.67	0.401
Prematurity	2.95	1.76–4.95	<0.001
Incomplete/unvaccinated status	2.78	1.94–3.98	<0.001
Any comorbidity	2.30	1.47–3.59	<0.001
Apnea†	7.91	5.25–11.93	<0.001
Cyanosis†	9.04	5.55–14.72	<0.001
Respiratory distress†	11.40	7.59–17.11	<0.001
Tachypnea	5.39	3.68–7.89	<0.001
Oxygen saturation <92%†	23.52	13.94–39.68	<0.001
Moist rales/crackles	3.15	2.17–4.57	<0.001
Radiological pneumonia	4.51	3.09–6.59	<0.001
Marked leukocytosis	8.63	5.57–13.37	<0.001
Lymphocytosis	4.42	3.04–6.42	<0.001
Elevated C-reactive protein	2.94	2.04–4.24	<0.001
Hypoalbuminemia	5.47	3.65–8.20	<0.001
Reduced immunoglobulin G	3.15	2.01–4.94	<0.001
Anemia	2.43	1.64–3.61	<0.001
WBC, per 5 × 10⁹/L increase	3.21	2.62–3.92	<0.001
Absolute lymphocytes, per 5 × 10⁹/L increase	7.79	5.42–11.18	<0.001
Albumin, per 5 g/L increase	0.33	0.26–0.42	<0.001
IgG, per 1 g/L increase	0.58	0.50–0.66	<0.001
Cough duration before admission, per day	1.01	0.98-1.05	0.532

Continuous variables are expressed in the same units as in the admission-based model (Table 7). Table 5 includes all variables entered in the multivariable models in Tables 6, 7. Univariable ORs for variables marked † are determined largely by the outcome definition. OR, odds ratio; CI, confidence interval. Each variable was analysed separately with severe pertussis as the dependent outcome. † Overlaps with a component of the severity definition.

### 3.6 Concurrent multivariable classification model of established severe disease

The other variables in the multivariable model were adjusted for and several variables were associated with severe pertussis (Table 6). This model incorporates the outcome definition so should be interpreted as a concurrent, rather than predictive, classification model for established severe disease. Variables included in this concurrent classification model were prespecified based on clinical relevance, prior literature, and their relationship to the predefined severity classification, rather than selected solely on univariable p-values. Children aged <6 months had higher odds of severe disease (adjusted OR 2.41, 95% CI 1.46–3.98, *p* = 0.001), as did those with a history of prematurity (adjusted OR 2.08, 95% CI 1.14–3.80, *p* = 0.017) and incomplete or absent vaccination (adjusted OR 1.79, 95% CI 1.15–2.80, *p* = 0.010). The association between incomplete/absent vaccination and severe disease was estimated after adjustment for age and other multivariable model variables, although this does not eliminate potential residual confounding due to age-dependent vaccination opportunities. Among clinical and laboratory factors, respiratory distress showed a strong association with severity (adjusted OR 4.21, 95% CI 2.61–6.80, *p* < 0.001), followed by marked leukocytosis (adjusted OR 4.37, 95% CI 2.59–7.37, *p* < 0.001) and apnea (adjusted OR 3.06, 95% CI 1.88–4.99, *p* < 0.001). Because both respiratory distress and apnea are part of the outcome definition, the adjusted ORs for these two outcomes are partly determined by the classification rule and should not be interpreted as independent risk factors; they may also inflate the adjusted estimates of the other outcomes in this model. The same was observed for radiological pneumonia, hypoalbuminemia and a decrease in immunoglobulin G in this model.

**Table 6:** Concurrent multivariable classification model of established severe pertussis (includes outcome-defining variables).

Predictor	Adjusted OR	95% CI	*p*-value
Age <6 months	2.41	1.46–3.98	0.001
Prematurity	2.08	1.14–3.80	0.017
Incomplete/unvaccinated status	1.79	1.15–2.80	0.010
Apnea†	3.06	1.88–4.99	<0.001
Respiratory distress†	4.21	2.61–6.80	<0.001
Radiological pneumonia	2.02	1.27–3.22	0.003
Marked leukocytosis	4.37	2.59–7.37	<0.001
Hypoalbuminemia	2.48	1.48–4.15	0.001
Reduced immunoglobulin G	1.71	1.01–2.89	0.046

Adjusted ORs were estimated using binary logistic regression. The model included age, prematurity, vaccination status, comorbidity, apnea, respiratory distress, radiological pneumonia, marked leukocytosis, hypoalbuminemia, and reduced immunoglobulin G. OR, odds ratio; CI, confidence interval. † Overlaps with a component of the severity definition; interpret as a marker of established severe disease. Because apnea and respiratory distress are components of the severity definition, their adjusted ORs represent concurrent classification of established severe disease and should not be interpreted as independent risk factors or prognostic predictors.

Figure 2 visually summarises the adjusted associations in the concurrent classification model; it is not a model of independent predictors.

**Figure 2:**
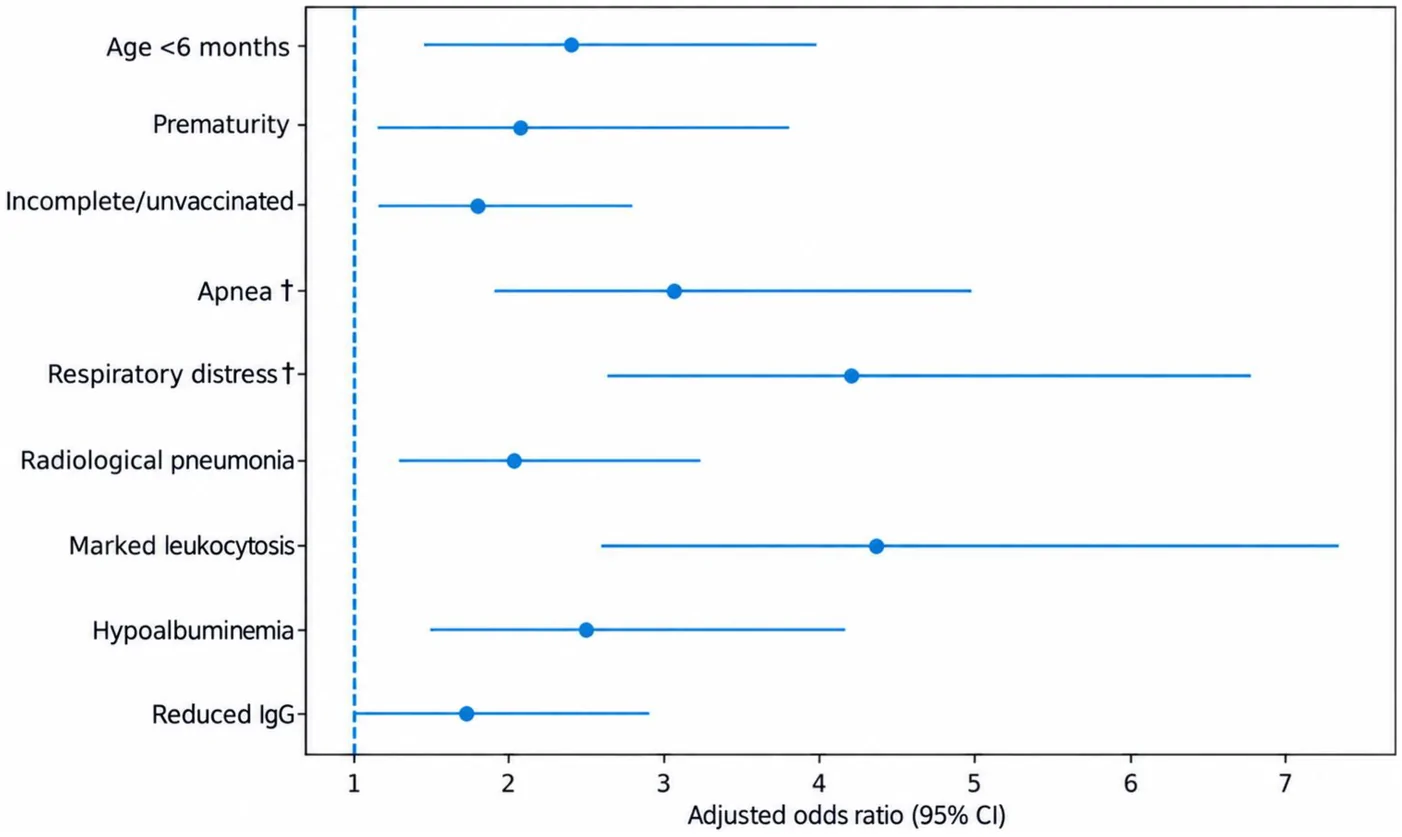
Forest plot of the concurrent multivariable classification model of established severe pertussis points represent adjusted odds ratios, and horizontal lines represent 95% confidence intervals. The vertical reference line indicates an odds ratio of 1.0. Values to the right of the reference line indicate an increased adjusted likelihood of severe pertussis. † Overlaps with the severity definition. Apnea and respiratory distress are components of the outcome definition; their ORs should not be interpreted as independent risk factors.

### 3.7 Admission-time severity classification model

A total of 104 children (66.2%) had already met at least one criterion for severe disease at the index time, 41 children (26.1%) had their first severe-disease event after the index time, and 12 children (7.6%) could not be reliably classified. Therefore, most of the severe cases met the outcome definition at or before the index time. A separate multivariable model restricted to information available at the index time was developed; this model included demographic, perinatal, vaccination, and comorbidity characteristics and admission laboratory values, but excluded apnea, respiratory distress, respiratory failure, respiratory support, intensive care admission, and other in-hospital outcome variables. In this model, age <6 months remained independently associated with severe pertussis (AOR 3.17, 95% CI 1.75–5.74; *p* < 0.001), as did incomplete-for-age or absent vaccination (AOR 2.59, 95% CI 1.42–4.70; *p* = 0.002). Higher WBC was associated with increasing odds of severe disease (AOR 3.10 per 5 × 10⁹/L increase, 95% CI 2.34–4.12; *p* < 0.001), as was a higher absolute lymphocyte count (AOR 7.09 per 5 × 10⁹/L increase, 95% CI 4.33–11.60; *p* < 0.001). Because lymphocytes form part of the total WBC, collinearity between these two variables was assessed; it was acceptable (VIF 1.19 for WBC and 1.13 for absolute lymphocyte count; Pearson r = 0.27), and all VIFs in the model were ≤3.56 (Supplementary Table S1). After patient-level verification of the laboratory records, the overall Spearman correlation between WBC and ALC was modest (*ρ* = 0.247), with similarly modest age-stratified correlations. This pattern indicates that variation in total WBC was not determined solely by variation in lymphocyte count across the pediatric age range, with age-stratified correlations of [*ρ* = 0.288] for ages 1–4 months, [*ρ* = 0.263] for ages 6–11 months, [*ρ* = 0.153] for ages 1–4 years, and [*ρ* = 0.117] for ages 5–13 years. Adjusted Odds Ratios were used for the simultaneous inclusion of WBC and ALC in the primary admission-time model as this indicated a conditional association, rather than simple effect. The adjusted ORs for the sensitivity analyses were 3.21 per 5 × 10⁹/L increase for WBC alone and 7.79 per 5 × 10⁹/L increase for ALC alone. Both ALC and non-lymphocyte WBC (WBC - ALC) were entered together, with the adjusted ORs of 25.68 and 3.28 respectively. These analyses confirmed the persistence of these associations and allowed a more interpretable decomposition of total leukocytes.

Conversely, higher serum albumin (AOR 0.41 per 5 g/L increase, 95% CI 0.29–0.58; *p* < 0.001) and higher IgG (AOR 0.64 per 1 g/L increase, 95% CI 0.52–0.79; *p* < 0.001) were associated with lower odds of severe pertussis (Table 7). In a sensitivity analysis using age-specific low-IgG status instead of continuous IgG concentration, the direction of association remained consistent, with age-specific low IgG associated with higher odds of severe pertussis (adjusted OR 2.85, 95% CI 0.86–9.44; *p* = 0.087) after adjustment for age, sex, prematurity, vaccination status, WBC, absolute lymphocyte count, and serum albumin. Although the confidence interval was wider and the association did not reach conventional statistical significance, the effect direction was consistent with the primary continuous-IgG analysis.

**Table 7:** Admission-time multivariable model for classification of severe pertussis.

Variable	Adjusted OR	95% CI	P value
Age <6 months	3.17	1.75–5.74	<0.001
Male sex	1.38	0.75–2.51	0.300
Prematurity	3.53	0.72–17.39	0.121
Incomplete/unvaccinated	2.59	1.42–4.70	0.002
Underlying comorbidity	0.85	0.20–3.59	0.828
WBC, per 5 × 10⁹/L increase	3.10	2.34–4.12	<0.001
Absolute lymphocytes, per 5 × 10⁹/L increase	7.09	4.33–11.60	<0.001
Albumin, per 5 g/L increase	0.41	0.29–0.58	<0.001
IgG, per 1 g/L increase	0.64	0.52–0.79	<0.001

AOR, adjusted odds ratio; CI, confidence interval; WBC, white blood cell count; IgG, immunoglobulin G. The model included only demographic, historical, and laboratory variables available at the index time (first admission blood sample); some children may already have met severe-pertussis criteria at that time. This model was restricted to variables available at the index time and was developed for admission-time classification of severe pertussis. Do not interpret its discrimination as prognostic prediction of subsequent clinical deterioration. An OR >1 indicates higher odds of severe pertussis, whereas an OR <1 indicates lower odds as the corresponding continuous variable increases. Apparent AUC 0.961 (95% CI 0.947–0.975); optimism-corrected AUC 0.957 (1,000 bootstrap resamples); Hosmer–Lemeshow *χ*^2^ = 5.45, *p* = 0.71; VIF 1.19 for WBC and 1.13 for absolute lymphocytes.

No statistically significant independent association was detected for sex, prematurity or underlying comorbidity after adjustment; the confidence interval for prematurity was wide (0.72–17.39). The apparent AUC of the admission-based model was 0.961 (95% CI 0.947–0.975; optimism-corrected AUC 0.957 after 1,000 bootstrap resamples). The AUC of 0.961 reflects discrimination between children ultimately classified as severe and non-severe using information available at admission; it should not be interpreted as prediction of subsequent clinical deterioration. At a threshold of 0.50, sensitivity was 75.2%, specificity 95.8%, overall accuracy 91.3%, and PPV 83.1%, while NPV was 93.3%. The Hosmer–Lemeshow test did not show poor fit (*χ*^2^ = 5.45, df = 8, *p* = 0.71); the calibration plot had a calibration intercept of [0.000] and calibration slope of [1.000] (optimism-corrected calibration slope = [0.935]). The restricted cubic spline plots showed no evidence of non-linearity in the continuous predictor variables, and modelling age as a continuous variable yielded similar values (AUC of 0.958).

When serum IgG was excluded from the sensitivity analysis, the simplified admission-time model remained the same as above, including age <6 months, sex, prematurity, vaccination status, underlying comorbidity, WBC, absolute lymphocyte count, and serum albumin. The apparent AUC was 0.956 (95% CI 0.941–0.972), with an optimism-corrected AUC of 0.952 after 1,000 bootstrap resamples. At a probability threshold of 0.50, sensitivity, specificity, accuracy, positive predictive value, and negative predictive value were 71.3%, 96.5%, 91.0%, 84.8%, and 92.4%, respectively. The apparent calibration intercept was ∼0, the calibration slope was ∼1.00, and the optimism-corrected calibration slope was ∼0.94. Consequently, excluding IgG would lead only to a slight decrease in apparent discrimination compared with the full admission-time model (Table 8). Further adjustment for C-reactive protein, feeding difficulty, alanine aminotransferase and creatinine led to an AOR of [0.43 per 5 g/L (95% CI 0.28-0.67)]. In a sensitivity analysis with additional adjustment for cough duration before admission, the estimates were essentially unchsanged (e.g., AOR 3.25 for age <6 months, 3.13 per 5 × 10⁹/L WBC and 7.19 per 5 × 10⁹/L lymphocytes), cough duration before admission itself was not associated with severity (AOR 0.98 per day, 95% CI 0.92–1.04; *p* = 0.413), and the AUC was 0.962 (Supplementary Table S1).

**Table 8:** Simplified admission-time multivariable model excluding serum igG.

Variable	Adjusted OR	95% CI	p-value
Age <6 months	3.33	1.88–5.91	<0.001
Male sex	1.29	0.72–2.29	0.388
Prematurity	2.70	0.58–12.62	0.208
Incomplete/unvaccinated	2.83	1.59–5.04	<0.001
Underlying comorbidity	1.09	0.27–4.40	0.904
WBC, per 5 × 10⁹/L increase	3.23	2.59–4.20	<0.001
Absolute lymphocytes, per 5 × 10⁹/L increase	7.46	4.94–11.24	<0.001
Albumin, per 5 g/L increase	0.39	0.31–0.48	<0.001

The coefficients above are based on the same admission-time model specification as Table 7, with IgG removed. WBC and ALC are entered simultaneously; therefore, their ORs represent conditional associations. Apparent AUC = 0.956 (95% CI 0.941–0.972); optimism-corrected AUC = 0.952 after 1,000 bootstrap resamples. Apparent calibration intercept ≈0.00; calibration slope = 1.00; optimism-corrected calibration slope ≈0.94.

### 3.8 Apparent discriminatory performance of the classification models

Table 9 shows the apparent discriminatory performance of the classification models in two analytically distinct groups: classification models that incorporate clinical variables which overlap with severe disease as defined above (concurrent models), and the model that classifies patients at the time of admission based on variables available at the time of the index admission (admission-time severity classification). These groups answer the different analyses questions and should not be compared directly as model performance estimates. The demographic model showed moderate discrimination (AUC 0.71 [95% CI 0.66–0.76]), with sensitivity 68.8% and specificity 65.5%. The clinical model had an AUC of 0.83 (95% CI 0.79–0.87), sensitivity of 78.3%, and specificity of 76.6%. The laboratory model had an AUC of 0.81 (95% CI 0.77–0.86); differences between the sequential models were not formally tested.

**Table 9:** Discriminatory performance of concurrent classification and admission time severity classification models.

Model	AUC	95% CI	Sensitivity, %	Specificity, %	Accuracy, %
Panel A. Concurrent classification models					
Demographic classification model	0.71	0.66–0.76	68.8	65.5	66.2
Clinical classification model	0.83	0.79–0.87	78.3	76.6	77.0
Laboratory classification model	0.81	0.77–0.86	74.5	78.2	77.4
Integrated clinical–laboratory classification model	0.89	0.86–0.92	83.4	82.1	82.4
Panel B. Admission-time severity classification model					
Admission-time severity classification model	0.961	0.947–0.975	75.2	95.8	91.3

AUC, area under the receiver operating characteristic curve; CI, confidence interval. The model in Panel A is designed for a different analytical question than the model in Panel B, and cannot be directly compared to the latter. Severe pertussis (as defined by the operational definition) is defined by clinical variables in the concurrent classification models, which could lead to outcome-information leakage. The admission time model was limited to variables measured at the predefined admission time, and was used to classify severe pertussis at admission, not to predict clinical deterioration. The 95% CI was calculated by using the DeLong method for the admission time model; the optimism corrected AUC after 1000 bootstrap resamples was 0.957.

The models developed were the demographic, clinical, laboratory, and integrated clinical–laboratory models, which were analysed concurrently; the apparent AUCs of these models may be influenced by leakage of outcome information, and their apparent AUCs are described, but not compared statistically, and were optimism-corrected using 1,000 bootstrap resamples, with an apparent AUC of 0.961 (95% confidence interval [CI] 0.947-0.975) for the admission-time severity classification model, which used only variables available at the prespecified index time.

As shown in Figure 3, the concurrent classification models and the admission-time severity classification model are presented in separate panels to emphasize their distinct analytical roles.

**Figure 3:**
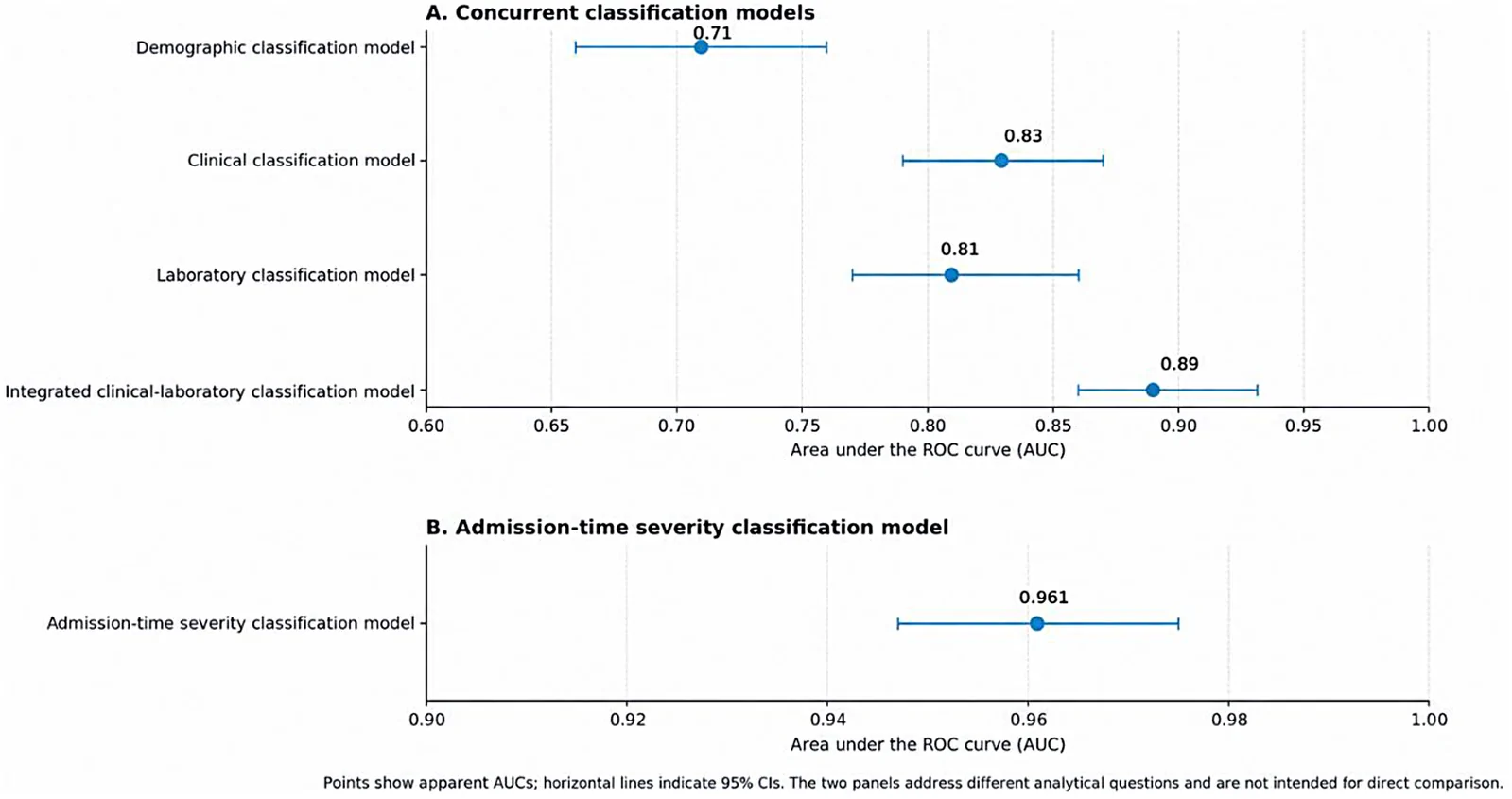
Discriminatory performance of concurrent classification and admission-time severity classification models.The points represent apparent AUCs and the horizontal lines represent 95% CIs. Panel **A** presents the concurrent classification models, which include overlapping clinical variables with the operational definition of severe pertussis and may therefore be confounded by leakage of outcome information. Panel **B** shows the admission-time severity classification model, which limited the variables to demographic, historical, and admission laboratory variables prespecified at index time. The two panels provide different answers to the analytical questions and the AUCs are not necessarily directly comparable as indicators of predictive performance. The admission-time model was created to classify severe pertussis at admission rather than to predict clinical deterioration.

## 4 Discussion

The present study found that younger age, especially infants aged <6 months, prematurity, low birth weight, incomplete or absent vaccination, and underlying comorbidities were significantly associated with pertussis severity, whereas sex showed no statistically significant association. These findings are similar to those of Kang et al. (20), who found that most severe cases occurred in young children with incomplete vaccination. Likewise, Guarnieri et al. (21) reported that low vaccination rates, younger age, and prematurity were significant characteristics associated with severe disease. The higher prevalence of comorbidities, such as congenital heart disease, chronic respiratory disorders, and neurological diseases among severe cases in the present study is also consistent with that of Gatto et al. (22), who reported that underlying comorbidities were associated with severe pertussis. Comorbidity was, however, not independently associated with severity after adjustment in the admission-based model. These findings as a whole highlight that infants, adverse perinatal histories, poor level of vaccination and underlying medical conditions are important risk factors for severe pertussis in children admitted to hospital.

In the present study, severe pertussis was linked to a significantly increased incidence of respiratory compromise (apnea, cyanosis, respiratory distress, tachypnea, tachycardia, chest retractions, hypoxemia, abnormal breath sounds and altered mental state). Because several of these features form part of the severity definition, these differences are expected and should be interpreted descriptively. No statistically significant differences were detected in the classic features of the disease (paroxysmal cough, inspiratory whoop, post-tussive vomiting or wheezing). This is consistent with Wang et al. (23), who found severe pertussis associated with prominent respiratory distress, apnea, cyanosis, and hypoxemia. Likewise, Ocal-Demir et al. (24) found that O2 desaturations, increased respiratory effort, altered consciousness, and respiratory distress were important clinical factors associated with severe disease requiring intensive management. The higher percentage of feeding problems and abnormal respiratory examination findings in the present study also supports Wang et al. (25), who found that progressive respiratory deterioration and systemic clinical deterioration characterise severe pediatric pertussis.

The present study showed severe pertussis was associated with significant changes in haematological, inflammatory, biochemical, and immunological parameters, such as an increase in WBC, lymphocytes, neutrophils, and platelets, in addition to elevated C-reactive protein, procalcitonin, and alanine aminotransferase, and a decrease in haemoglobin, serum albumin, and immunoglobulin G (IgG) levels. These findings are comparable to those of Guo et al. (26), who reported a strong correlation between leukocytosis, lymphocytosis, and high levels of inflammatory markers and disease severity, along with better classification of severe pertussis cases. Similarly, the increased frequency of hypoalbuminemia, anaemia, and decreased immunoglobulin G reported in the present study is consistent with the findings of Zhang et al. (27). In severe cases, however, the low IgG should be interpreted with caution: IgG is mostly maternally derived and physiologically decreases in the first months of life (12); severe cases were younger; and capillary leak or protein loss may also lead to low levels of IgG. Accordingly, the observed association between lower IgG concentration and severe pertussis should be interpreted as an observational association rather than evidence that low IgG causes severe disease. We cannot differentiate between these explanations with our data. In addition, haematological and biochemical markers also showed clinical significance in risk stratification, as Gao et al. (28) found that these markers, in combination, discriminated better for severe pertussis.

Hypoalbuminemia should be interpreted as a marker associated with severe pertussis rather than as an independent causal risk factor. The observed association may reflect reduced intake, inflammation, fluid shifts, hepatic or renal dysfunction, protein loss, or other aspects of established illness. Because these factors were not comprehensively captured, residual confounding cannot be excluded. The 42.0% prevalence in the severe group is comparable with the mean albumin level (34.8 ± 5.1 g/L) as the lower reference limits in the institution (set at <36 g/L for children aged 1–<12 months and <38 g/L for children aged 1–13 years) indicate even mild decreases as hypoalbuminemia, and this agrees with the albumin levels in children reported in published laboratory studies. Low albumin levels are associated with a lack of oral intake, fluid overload, malnourishment, liver or kidney failure, protein-losing disorders and known severe illness, as well as inflammation. The association weakened (but was not eliminated) after further adjustment for C-reactive protein, feeding difficulty, alanine aminotransferase, and creatinine; albumin was measured on admission, which further limits the impact of in-hospital fluid therapy. No systematic information was collected on nutritional status, oedema, fluid balance and protein-loss conditions, and residual confounding cannot be ruled out, as our results suggest an association with severity only.

The present study showed that severe pertussis was accompanied by significantly more radiological involvement (pneumonia, pulmonary consolidation, atelectasis, multilobar involvement), respiratory failure, need for intensive care, advanced respiratory support, extended hospital stays and in-hospital death. This aligns with previous studies indicating that the extent of lung involvement and respiratory complications are significant factors in the severity and clinical outcome of children with pertussis admitted to hospital (29). Similarly, higher rates of mechanical ventilation and critical care admission, and higher mortality in severe cases, support the multinational study by Alghounaim et al. (30), which found that severe respiratory impairment is strongly linked to a worse prognosis and increased healthcare resource utilisation in paediatric pertussis.

The present univariable logistic regression analysis revealed that several demographic, clinical, radiological and laboratory parameters were associated with severe pertussis. Younger age (<6 months) and prematurity were significantly associated, while incomplete or absent vaccination was independently associated with higher odds of severe pertussis in the adjusted analysis. Because vaccination status is closely related to age and eligibility for vaccine doses, this association should not be interpreted as a causal estimate from the present retrospective analysis (31). Hypoxemia, respiratory distress, cyanosis, and apnea showed the strongest associations, although this partly reflects their inclusion in the severity definition. Similarly, radiological pneumonia and abnormal laboratory results such as severe leukocytosis, lymphocytosis, high C-reactive protein, hypoalbuminemia, low immunoglobulin G and anaemia were significantly correlated with severe disease. These results are consistent with previous studies indicating that inflammatory and haematological abnormalities, particularly hyperleukocytosis, are significant indicators of pertussis severity and poor outcome (32, 33). In general, early detection of these clinical and laboratory markers may help better risk-stratify children with severe pertussis and support timely management.

In the multivariable model, several factors remained independently associated with severe pertussis. Prematurity, incomplete-for-age vaccinations, and younger age (<6 months) remained independently associated with severe pertussis. Because vaccination status depends on age and the opportunity to receive the recommended doses, interpret the observed association between incomplete-for-age vaccination and severe pertussis with caution. This hospital-based study did not use a design appropriate for assessing vaccine effectiveness (e.g., a test-negative design), and vaccination status was not a primary exposure, so the results cannot be interpreted as a vaccine-effectiveness assessment. Both respiratory distress and apnea had strong adjusted associations but are part of the severity definition and are more likely markers of established severe disease than predictors. Marked leukocytosis remained independently associated with severity, consistent with prior reports linking hyperleukocytosis to poor outcome (6, 32, 33). Additionally, plasma levels of radiological pneumonia, hypoalbuminemia, and reduced immunoglobulin G were all correlated with severity on this concurrent model (all are included as components of the outcome) and thus are observationally associated with severe disease, but do not provide any mechanistic or causal pathway. These results corroborate recent research showing that age, respiratory symptoms, immune abnormalities, and inflammatory markers better predict severe pertussis and complications (34–36). The integrated clinical–laboratory model included variables that overlap with the operational definition of severe pertussis. Its apparent discrimination is therefore partly attributable to outcome-information leakage and should be interpreted as concurrent classification of established severe disease rather than prognostic prediction.

Factors known to be important for established severe disease are not clinically identical to variables appropriate for severity at admission. One of the main problems in understanding the admission-time model is the temporal relationship between index time and severe disease. The 104 children (66.2%) with severe disease by the index time met one or more of the severe disease criteria, while 41 children (26.1%) met their first criteria for severe disease after the index time, and 12 (7.6%) could not be reliably determined as to the timing of first severe disease. Therefore, the AUC of 0.961 should be understood as a measure of discrimination in classifying children admitted to the hospital as severe or non-severe based on information available at hospital admission, rather than as a measure of discrimination for severe disease at or around the index time. The model therefore characterizes admission-time attributes associated with severe disease in this study sample rather than predicting subsequent clinical deterioration.

Another factor that supports the practicality of the admission-time model is test availability. Most hospitals have routine access to WBC, differential count, and albumin results within a few hours, whereas serum IgG results may not be readily available in most hospitals, including primary and secondary care in LMIC countries, and may take longer than the initial triage period. In this dataset, IgG was measured in all 722 children; Serum IgG included in the standardized admission immunologic evaluation of children who were admitted to the study centre with a confirmed diagnosis of pertussis. A simplified admission-time model excluding IgG retained strong apparent discrimination (AUC 0.956; 95% CI 0.941–0.972), with an optimism-corrected AUC of 0.952 after 1,000 bootstrap resamples. This was only modestly lower than the full model (AUC 0.961), supporting the potential practical relevance of an IgG-free model when serum IgG is not rapidly available during initial assessment.

Among the demographic, clinical, laboratory, and integrated models, the integrated model showed the best apparent AUC (0.89), but this does not imply better prognostic performance. Components of the outcome definition, such as apnea and respiratory distress, are included in the clinical and integrated models and thus likely inflate AUCs due to outcome-information leakage; these models describe known severe disease, do not constitute prognostic models for subsequent deterioration. None of the five models was superior to the others, as they are not sequential and no paired tests were available to compare AUCs. Results are consistent with recent studies indicating that models using clinical parameters and blood markers demonstrate stronger discrimination for severe pertussis, and may assist in characterising children with greater severity at or around admission (37, 38). The admission-time model provides a classification framework based on demographic, historical, and laboratory variables available at the index time. Its clinical utility for identifying children who are initially non-severe but subsequently deteriorate remains uncertain and requires prospective validation with a clearly defined index time and prospectively recorded severe-event timing.

### 4.1 Strengths of the study

The study has several strengths. It included a relatively large number of children confirmed to have pertussis by laboratory tests, providing enough events (157 severe cases) to examine factors associated with illness severity. A full spectrum of demographic, clinical, radiological, haematological, biochemical and immunological parameters was evaluated, enabling the disease to be assessed in multiple dimensions. Multivariable logistic regression identified factors independently associated with severity and adjusted for measured confounders. We also separated admission-available variables from features of established severe disease and internally validated the admission-based model by bootstrapping. We also redrew the model comparison without suggesting sequential model improvement and clearly defined the models with and without outcome-defining variables.

### 4.2 Limitations of the study

Although these are strengths, some things should be noted. The retrospective design relied on routinely collected medical records and may have introduced selection and information bias, and some variables were incompletely documented. Of these, 30 records were excluded because essential clinical, laboratory or severity data was missing and 18 were excluded because insufficient documentation was found to confirm eligibility. But since patient-level information from these excluded records was not saved in the analysis dataset, their clinical and laboratory parameters could not be analyzed against the included cohort. Thus, the exclusion of complete cases cannot be excluded as a potential source of selection bias. Because the study was carried out in a single tertiary care centre, the results may not be widely applicable to other healthcare centres and/or geographic areas with different patient demographics or resources. Furthermore, some potentially relevant factors (e.g., maternal vaccination status, socioeconomic factors and long-term clinical outcomes) were not gathered for analysis.

A major limitation is that the model reflects the temporal interpretation at the time of admission. Of 157 children categorized as having severe pertussis, 104 (66.2%) had at least one severe event prior to or at the same time as the index event, 41 (26.1%) had their first severe event after the index event, and for 12 (7.6%) the timing was not reliably established. The model built on retrospective records cannot be considered a model of subsequent deterioration, but rather a classification model for severity, since the timing of events that became severe relative to the index time was not fully reconstructed. The observed AUC thus reflects discrimination between children who ended up in the severe category and those who did not, rather than the model's ability to predict subsequent deterioration in children originally assigned to the non-severe category.

The classification models showed good apparent discrimination in the study population, but only the admission-based model was internally validated, and neither was validated in independent populations, so they are not immediately applicable in clinical practice. Because the secondary analysis used demographic, clinical-history, and laboratory variables assessed at admission, the retrospective design nonetheless limits precise determination of the temporal relationship between these baseline measurements and subsequent clinical deterioration. A further consequence is that children who had already fulfilled severe-disease criteria at the index time could not be completely separated from those whose first qualifying severe event occurred later in the clinical course; the model was therefore an admission-time severity classification model, not a model for subsequent worsening. Vaccination status was classified by age-appropriate completion categories rather than detailed dose-by-dose vaccination histories; therefore, residual confounding by age-related vaccination opportunity and differences in the timing of individual doses cannot be completely excluded.

Several other limitations should be noted. First, the severity definition includes apnea, respiratory distress, cyanosis and hypoxemia, which were also candidate variables, so their associations with severity are circular; the multivariable and integrated models were therefore interpreted as concurrent classification of established severe disease, and their AUCs are likely inflated by outcome-information leakage. Second, the study did not include a non-pertussis control group; therefore, the patterns of laboratory variables observed should not be considered specific to pertussis. Third, the study period (2020-2025) overlapped with the Covid-19 pandemic and the resurgence of pertussis, which may have led to changes in influenza and macrolide resistance in circulation and in clinical practice; a calendar year of admission is not available in the analysis data set, and residual temporal confounding cannot be excluded. Fourth, although all included participants had documented laboratory-confirmed pertussis, the analysis dataset did not retain the individual laboratory confirmation method for each case. Therefore, the proportion confirmed specifically by PCR could not be reliably reconstructed, and a sensitivity analysis restricted to PCR-confirmed cases was not possible. Fifth, between-group comparisons presented in Tables 1–4 are not adjusted for multiple testing and should be considered as exploratory. Sixth, the integrated model dichotomised several laboratory factors, discarding information. Further, the age-specific approach of categorizing IgG did not yield statistically significant results and the confidence intervals were wider, although the direction of the association with severe pertussis remained the same; thus, the IgG association should be viewed with caution, especially when the strong age dependency of IgG concentrations in infancy is taken into account. Seventh, in some areas, serum IgG might not be available routinely or quickly enough for an admission-based model to be used as the first line of triage. Eighth, nutritional status, oedema, fluid balance and hepatic, renal or protein losing conditions were not adequately reflected and hence the relationship of low albumin with severity may be confounded.

### 4.3 Future recommendations

Future prospective, multicenter studies should capture the precise time of each severe event in relation to a specific time point (predefined index time) to build a true prognostic model for new severe events post-admission and to externally validate the model in diverse pediatric populations. Further model development could benefit from additional biomarkers (molecular or immunological markers), longer follow-up, and information on maternal vaccination. Finally, future research should assess whether prospectively developed and externally validated prognostic models can support clinical decision-making, early therapeutic interventions, health care resource utilisation, and patient outcomes. This would provide stronger evidence on whether validated prognostic tools could be incorporated into routine care for children with pertussis and help reduce morbidity and mortality associated with the disease.

## 5 Conclusion

The study identified several factors associated with severe pertussis in children who were hospitalised, including being under 6 months of age, prematurity, incomplete-for-age or absent vaccination, radiological pneumonia, marked leukocytosis, hypoalbuminemia and decreased immunoglobulin G levels; apnea and respiratory distress were also strongly associated but overlap with the definition of severe disease. Because the outcome definition is part of the integrated clinical–laboratory model, it incorporates elements of the outcome and the apparent discrimination may be more than warranted. A model using only demographic and laboratory factors available at admission has high apparent discrimination for classifying severe disease at or around admission, however, it should not be interpreted as a model for predicting subsequent clinical deterioration, and serum IgG might not be measured during rapid triage in all clinical settings. Hypoalbuminemia should be interpreted as a marker associated with severe disease rather than as a causal factor. The study identified several demographic and laboratory characteristics associated with severe pertussis, including age under 6 months, prematurity, incomplete or absent vaccination, radiological pneumonia, marked leukocytosis, hypoalbuminemia, and lower IgG levels. Further prospective multicenter studies with a prespecified index time and prospectively recorded timing of severe events are needed to determine whether these admission-time characteristics can identify initially non-severe children who subsequently deteriorate and to assess their generalizability across clinical settings.

## Data Availability

The raw data supporting the conclusions of this article will be made available by the authors, without undue reservation.
