# Supplementary material for: Integrated clinical and laboratory assessment for severity classification in pediatric pertussis
Source: Front Med (Lausanne). 2026 Oct 21;13:1961021. doi: 10.3389/fmed.2026.1961021 (PMC13730875; doi:10.3389/fmed.2026.1961021)
Supplement: Supplementary file 1 [file Table1.docx]

**Supplementary Material**

**Integrated Clinical and Laboratory Assessment of Risk Factors for Disease Severity in Pediatric Pertussis**

**Supplementary Table S1. Admission-based model: sensitivity analysis adjusting for cough duration, and variance inflation factors**

| **Variable** | **Main model, AOR (95% CI)** | **+ Cough duration, AOR (95% CI)** | **VIF** |
| --- | --- | --- | --- |
| Age <6 months | 3.17 (1.75–5.74) | 3.25 (1.79–5.90) | 1.07 |
| Male sex | 1.38 (0.75–2.51) | 1.37 (0.75–2.51) | 1.00 |
| Prematurity | 3.53 (0.72–17.39) | 3.56 (0.73–17.48) | 3.56 |
| Incomplete/unvaccinated | 2.59 (1.42–4.70) | 2.64 (1.45–4.82) | 1.04 |
| Underlying comorbidity | 0.85 (0.20–3.59) | 0.87 (0.21–3.67) | 3.55 |
| WBC, per 5 ×10⁹/L | 3.10 (2.34–4.12) | 3.13 (2.36–4.16) | 1.19 |
| Absolute lymphocytes, per 5 ×10⁹/L | 7.09 (4.33–11.60) | 7.19 (4.38–11.81) | 1.13 |
| Albumin, per 5 g/L | 0.41 (0.29–0.58) | 0.41 (0.29–0.59) | 1.12 |
| IgG, per 1 g/L | 0.64 (0.52–0.79) | 0.64 (0.52–0.79) | 1.08 |
| Cough duration, per day | – | 0.98 (0.92–1.04) | – |
| Apparent AUC (95% CI) | 0.961 (0.947–0.975) | 0.962 | – |

AOR, adjusted odds ratio; CI, confidence interval; VIF, variance inflation factor (calculated for the main model); WBC, white blood cell count; IgG, immunoglobulin G; AUC, area under the receiver operating characteristic curve. Both models were fitted by binary logistic regression in all 722 children (157 severe cases). The sensitivity model adds cough duration before admission (days) to the main model. Main model calibration: Hosmer–Lemeshow χ² = 5.45, df = 8, p = 0.71. Internal validation: optimism-corrected AUC 0.957 (1,000 bootstrap resamples). The 95% CI of the AUC was calculated by the DeLong method.
