# Supplementary material for: Integrated clinical and laboratory assessment for severity classification in pediatric pertussis
Source: Front Med (Lausanne). 2026 Oct 21;13:1961021. doi: 10.3389/fmed.2026.1961021 (PMC13730875; doi:10.3389/fmed.2026.1961021)
Supplement: Supplementary file 2 [file Table2.docx]

**Supplementary Table S2. Distribution and overlap of individual components of the composite severe-pertussis outcome among severe cases (n = 157)**

| **Component** | **Severe cases, n (%)** |
| --- | --- |
| Respiratory distress | 110 (70.1) |
| Pediatric ICU admission | 126 (80.3) |
| Respiratory failure | 91 (58.0) |
| Clinically documented apnea | 81 (51.6) |
| Oxygen saturation <92% | 80 (51.0) |
| Cyanosis | 60 (38.2) |
| Invasive mechanical ventilation | 55 (35.0) |
| Noninvasive respiratory support | 48 (30.6) |
| Pulmonary hypertension | 21 (13.4) |
| Neurological complications | 20 (12.7) |
| In-hospital mortality | 13 (8.3) |
